# Water-Soluble Vitamins (Riboflavin, Niacin, Pantothenic Acid) in Dogs with Chronic Liver Disease vs. Healthy Controls

**DOI:** 10.3390/vetsci12090877

**Published:** 2025-09-11

**Authors:** Verena Habermaass, Aurora Cogozzo, Francesco Bartoli, Valentina Vitelli, Rebecca Dini, Veronica Marchetti

**Affiliations:** 1 Department of Veterinary Sciences, Veterinary Teaching Hospital “Mario Modenato”, University of Pisa, Via Livornese Lato Monte, San Piero a Grado, 56122 Pisa, Italy; verena.habermaass@phd.unipi.it (V.H.); veronica.marchetti@unipi.it (V.M.); 2 Department of Translational Research and New Technologies in Medicine and Surgery, University of Pisa, Via Roma 67, 56126 Pisa, Italy; 3 Department of Surgical, Medical, Molecular Pathology and Critical Area, University of Pisa, Via Roma 67, 56126 Pisa, Italy

**Keywords:** vitamins, canine chronic liver disease, canine hepatic disease, water-soluble, riboflavin, B2, niacin, B3, pantothenic acid, B5

## Abstract

Background: Water-soluble vitamin deficiencies are common in human chronic liver disease (CLD) due to impaired metabolic pathways. Vitamins B2 (riboflavin), B3 (niacin), and B5 (pantothenic acid) assume critical roles in hepatic and lipid metabolism and may exert hepatoprotective effects. In canine CLD, data beyond cobalamin are sparse, and no guidelines currently endorse B-vitamin supplementation. Methods: This case–control study analysed 66 stored serum samples from client-owned dogs with CLD and 50 from healthy blood-donor dogs. CLD diagnosis required persistent (>2 months) elevation of at least two liver enzymes (ALP, GGT, AST, ALT) and ultrasonographic evidence of CLD. Serum vitamin concentrations were quantified by LC-MS/MS (ng/mL). Results: Vitamin B2 was significantly lower in CLD dogs versus controls (median 48.4 vs. 85.5 ng/mL; *p* = 0.002). No significant difference was observed in B3 levels (*p* = 0.25). Vitamin B5 concentrations were significantly higher in the CLD group (median 176.5 vs. 116.1 ng/mL; *p* = 0.003). Conclusions: Reduced B2 may reflect impaired hepatic processing or absorption in canine CLD. The presence of normal or elevated B3 and B5 may relate to alternative metabolic pathways. This constitutes the first study assessing B2, B3, and B5 in canine CLD, underscoring riboflavin’s potential interest in CLD dogs.

## Simple Summary

In humans, deficiencies in vitamins B2 (riboflavin), B3 (niacin), and B5 (pantothenic acid) are common in liver disease, as these vitamins support liver and fat metabolism. However, very little is known about these vitamins in dogs with chronic liver disease, and there are currently no official recommendations for vitamin supplementation in affected dogs. This study aimed to measure and compare blood levels of vitamins B2, B3, and B5 in dogs with chronic liver disease and in healthy dogs. Blood samples from 66 dogs with liver disease and 50 healthy donor dogs were analyzed. Diagnosis of liver disease was based on long-term increases in liver enzymes and ultrasound evidence of liver damage. Vitamin levels were measured using a highly accurate laboratory technique. The results showed that dogs with liver disease had much lower levels of vitamin B2 compared to healthy dogs. Vitamin B3 levels were similar in both groups, while vitamin B5 levels were higher in dogs with liver disease. These findings suggest that low levels of vitamin B2 may be linked to problems in how the liver absorbs or processes this vitamin. This study may help improve future nutritional support and treatment options for dogs living with chronic liver diseases.

## 1. Introduction

Deficiencies in water-soluble vitamins are frequently observed in human patients with end-stage liver disease and metabolic dysfunction-associated steatotic liver disease (MASLD) [1,2]. These deficiencies may arise from a multitude of metabolic alterations, including impaired intestinal absorption, reduced hepatic storage capacity, and dysfunctional enzymatic conversion—processes that are intricately dependent on normal liver function. The liver plays a central role in the metabolism, activation, and storage of several essential vitamins, particularly those belonging to the B-complex group. In the context of chronic liver disease (CLD) in humans, vitamin supplementation is often employed as an adjunctive therapeutic strategy to support residual hepatic function, mitigate oxidative stress, and optimise metabolic pathways disrupted by hepatocellular dysfunction [1,2].

Among the B vitamins, riboflavin (B2), niacin (B3), and pantothenic acid (B5) are of particular interest due to their indispensable roles in mitochondrial energy production, fatty acid oxidation, and the biosynthesis of coenzymes and neurotransmitters [3,4,5,6]. These vitamins are essential to hepatic and lipid metabolism and may exert hepatoprotective effects through a variety of mechanisms, including antioxidant activity, modulation of inflammatory responses, and enhancement of mitochondrial function [7,8].

Despite their well-established significance in human hepatology, the status of B vitamins in canine chronic liver disease remains largely unexplored. The current veterinary literature has focused predominantly on the evaluation of vitamin B12 (cobalamin) [9,10], with scant attention paid to the broader B-complex profile. Furthermore, no formal guidelines currently recommend supplementation of B2, B3, or B5 in dogs with chronic liver disease, highlighting a critical gap in clinical knowledge and practice.

In this context, the present study aimed to investigate and compare serum concentrations of vitamins B2, B3, and B5 in dogs diagnosed with CLD and in healthy control dogs, with the objective of identifying potential alterations in vitamin status that may inform future diagnostic, therapeutic, and nutritional interventions in veterinary hepatology.

## 2. Materials and Methods

This study was carried out in full compliance with the ethical principles outlined in the Declaration of Helsinki and received approval from the Ethics Committee of the University of Pisa (protocol number 41; approval date: 29 October 2020). Client-owned dogs presented to the Internal Medicine Service of the Veterinary Teaching Hospital at the University of Pisa between January 2021 and January 2023, and diagnosed with CLD, were prospectively enrolled.

The diagnosis of CLD was established based on a comprehensive assessment that included the patient’s medical history, physical examination, complete blood count, serum biochemistry, and abdominal ultrasonographic evaluation. Inclusion criteria required the persistence of elevated liver enzyme activities for longer than two months, with elevations observed in at least two of the following enzymes: alkaline phosphatase (ALP) > 250 U/L (reference interval: 45–250 U/L), gamma-glutamyl transferase (GGT) > 11 U/L (reference interval: 2–11 U/L), alanine aminotransferase (ALT) > 70 U/L (reference interval: 20–70 U/L), and aspartate aminotransferase (AST) > 40 U/L (reference interval: 15–40 U/L). In addition to biochemical abnormalities, diagnostic criteria required the presence of ultrasonographic findings consistent with chronic hepatobiliary disease [11]. These included: diffusely hyperechoic hepatic parenchyma, altered liver size and/or irregular hepatic margins, presence of nodular hepatic lesions suggestive of benign hyperplasia, gallbladder wall thickening with increased echogenicity and irregular contours, abnormal gallbladder contents such as mucocele formation, non-dependent biliary sludge, or cholelithiasis, chronic dilation of the intrahepatic biliary tree or common bile duct, and intrahepatic biliary mineralization. CLD dogs underwent measurement of baseline preprandial ammonia, bile acids (unless cholestatic) as part of the screening process, and those with increased concentrations were excluded. Dogs with ultrasonographic evidence of biliary tract abnormalities and increased bile acids were not excluded.

Among CLD dogs, cholestatic processes were identified in the presence of a biliary tract disease (BTD). BTD was diagnosed when at least two of the following laboratory abnormalities were concurrently present: ALP > 250 U/L, GGT) > 11 U/L, total bilirubin > 0.3 mg/dL (reference interval: 0.07–0.3 mg/dL), or cholesterol > 280 mg/dL (reference interval: 120–280 mg/dL), in association with at least one ultrasonographic alteration of the biliary tract [11]. The ultrasonographic features considered consistent with BTD included thickened, hyperechoic, and irregular gallbladder walls; abnormalities of gallbladder contents (e.g., mucocele, non-gravity-dependent biliary sludge, cholelithiasis); chronic dilatation of the intrahepatic biliary tree or of the common bile duct; and intrahepatic biliary mineralization. Based on this classification, dogs affected by chronic liver disease (CLD) were stratified into subgroups according to biliary tract involvement, specifically BTD and non-BTD. Within the framework of abdominal ultrasonography, the presence of concomitant chronic intestinal abnormalities—such as alterations in wall layering, echogenicity, or intestinal wall thickening—was systematically recorded in the examined dogs.

A control population consisting of clinically healthy blood-donor dogs was also included in the study. These animals were part of an established institutional donor program and were routinely subjected to a thorough pre-donation health screening protocol. This protocol comprised a complete physical examination, comprehensive hematological and biochemical profiling, and serological testing for vector-borne diseases, including Leishmaniosis and other common tick-borne diseases.

For all enrolled dogs, blood samples were obtained via jugular venipuncture following a 12 h fasting period. Samples intended for biochemical analysis were collected in serum-separating tubes. Within 15 min of collection, the blood samples were centrifuged, and the resulting serum was submitted for routine biochemical testing using an automated analyzer (Liasys, Assel SRL, Rome, Italy). Residual serum aliquots were transferred into Eppendorf tubes, frozen at −18 °C within 24 h, and subsequently stored at −80 °C for long-term preservation. None of the samples utilized in this study had been stored for longer than 24 months. In both study groups, serum samples used for B-vitamin quantification were derived exclusively from these surplus aliquots, routinely archived at −80 °C for research purposes.

Dogs with recent vitamin supplementation (<two months) or dogs receiving an unbalanced commercial or home-cooked diet were excluded. Dogs with significant comorbidities (chronic kidney disease, cardiac/oncologic/hematologic diseases) or acute hepatobiliary diseases (i.e., gallbladder mucocele rupture, biliary tract obstruction, acute hepatitis) with acute clinical signs were excluded.

As for the HPLC-MS/MS sample analysis, 50 µL of a 100 ng/mL ISTD mixture (Vitamin B2-^13^C_4_,^15^N_2_, Vitamin B3 ^13^C_6,_ Vitamin B5 ^13^C_3_,^15^N) and 400 µL of methanol were added to 50 µL of serum sample and vortexed. After a centrifugation of 20 min, at 4 °C, at 15,000 rpm, 50 µL of supernatant was transferred to a new 1.5 mL tube and dried under nitrogen flow, at 40 °C. Sample was reconstituted with 100 µL of water: methanol 90:10 (*v*/*v*) and injected for the analysis. Vitamin B2, B3, B5 were analysed and quantified by the HPLC-MS/MSsystem with an instrument layout consisting of a thermostated autosampler, a binary pump, and a column oven, all of them Agilent (Santa Clara, CA, USA) 1290 Infinity series, coupled to an AB Sciex (Vaughan, ON, Canada) API 4000 triple quadrupole mass spectrometer, equipped with a ESI Turbo-V Ion spray source. Chromatographic separation was performed by a reverse-phase column Zorbax SB-C18 StableBond Analytical 4.6 × 150 mm, 5 μm particle size (Agilent Corporation, Palo Alto, CA, USA), protected by a C18 3 mm ID security guard ULTRA cartridge, and eluents methanol and water, both containing 0.025 mM of acetic acid. Flow rate was 0.8 mL/min. The program of the HPLC-MS/MS run relative to solvent A was the following: 0.0–1.0 min at 7% (isocratic), 1–8.0 min from 7% to 100% (ramp), 8.0–9.5 min at 100% (isocratic), 9.5–10.0 min from 100% to 7% (ramp) and 10.0–12.0 min at 7% (equilibration). The HPLC-MS/MS column was thermostated at 40 °C, and the injection volume was 10 μL. The MS method was based on positive ion multiple reaction monitoring (MRM) mode: for each analyte, the transition with the highest signal-to-noise ratio was used as a quantifier, the others as qualifiers. Making use of optimized declustering potential (DP), collision energies (CEs), and collision exit potentials (CXPs), three transitions were monitored for each compound. Further operative parameters were set as follows: IonSpray voltage (IS), 3250 V; Gas Source 1 (GS1), 70 arbitrary units; Gas Source 2 (GS2), 60 arbitrary units; source Temperature (TE), 600 °C; Collision Gas (CAD) Nitrogen, 10 mPa, Curtain Gas (CUR) 10 arbitrary units; Prefilter (ST), −15.4 V; Focusing Lens 1 (IQ1), −10.6 V. Main MS parameters are summarised in Table 1. For the quantitative analysis, calibration curves containing stable isotope-labeled internal standards were prepared with 10 different dilution levels within the concentration range 0.06–10 ng/mL for vitamin B2, and a range of 0.3–50 ng/mL for vitamins B3 and B5. Details are reported in Table 1.

Statistical analysis was performed using GraphPad Prism 9. As data were non-normally distributed (Kolmogorov–Smirnov test), results are expressed as median (range), and differences between groups were evaluated using the Mann–Whitney U-test. Statistical significance was set at *p* < 0.05. To investigate whether serum levels of vitamins varied according to serum liver enzymes (ALP, GGT, AST, ALT) levels, Spearman’s Correlation test was used.

## 3. Results

### 3.1. Animals

A total of 66 dogs diagnosed with CLD, with a median age of 7 years (2–13 years), were included. In total, 37/66 (56%) were female (28 neutered, 9 intact) and 29/66 (44%) were male (15 neutered, 14 intact). The majority of dogs were mixed breed (n = 12; 18.2%), followed by Poodle (n = 5; 7.6%), Dachshund (n = 4; 6.1%), Cocker spaniel (n = 4; 6.1%), Yorkshire terrier (n = 4; 6.1%), Jack Russell terrier (n = 4; 6.1%), Maltese (n = 3; 4.5%), Cavalier King Charles spaniel (n = 3; 4.5%), Golden Retriever (n = 3; 4.5%), Labrador Retriever (n = 3; 4.5%), Shih Tzu (n = 2; 3.0%), West Highland White terrier (n = 2; 3.0%), Setter (n = 1; 1.5%), Zwergpinscher (n = 1; 1.5%), German Shepherd (n = 1; 1.5%), French bulldog (n = 1; 1.5%), Breton (n = 1; 1.5%), Boxer (n = 1; 1.5%), Chihuahua (n = 1; 1.5%), Beagle (n = 1; 1.5%), Boston terrier (n = 1; 1.5%), Flat Coated Retriever (n = 1; 1.5%), and Bull terrier (n = 1; 1.5%). The serum biochemical findings for CLD dogs are reported in Table 2. According to the biliary tract involvement subclassification, 32 out of 66 (48.5%) CLD dogs were diagnosed with BTD, whereas the remaining 34 were classified as non-BTD. With regard to chronic intestinal involvement detected by ultrasonography, 20 out of 66 (30.3%) dogs exhibited chronic ultrasonographic alterations of varying severity affecting one or more intestinal segments.

Fifty healthy controls (HC) were enrolled, with a median age of 5 years (2–8). Thirty-five were female (20 intact, 15 neutered), whereas the remaining 15 were male (9 intact, 6 neutered). According to the breed distribution, Mix-breed (n = 13; 26%), Golden Retriever (n = 7; 14%), Weimaraner (n = 5; 10%), Dobermann (n = 5; 10%), Bernese Mountain Dog (n = 3; 6%), Labrador Retriever (n = 3; 6%), Setter Gordon (n = 2; 4%), Italian Pointing Dog (n = 2; 4%), Newfoundland (n = 2; 4%), American Staffordshire Terrier (n = 1; 2%), Boxer (n = 1; 2%), Cane Corso (n = 1; 2%), Greater Swiss Mountain Dog (n = 1; 2%), Greyhound (n = 1; 2%), Belgian Shepherd (n = 1; 2%), Rhodesian Ridgeback (n = 1; 2%). The age did not statistically differ between CLD and HC groups (*p* = 0.67).

### 3.2. Analysis Results

**Figure 1:**
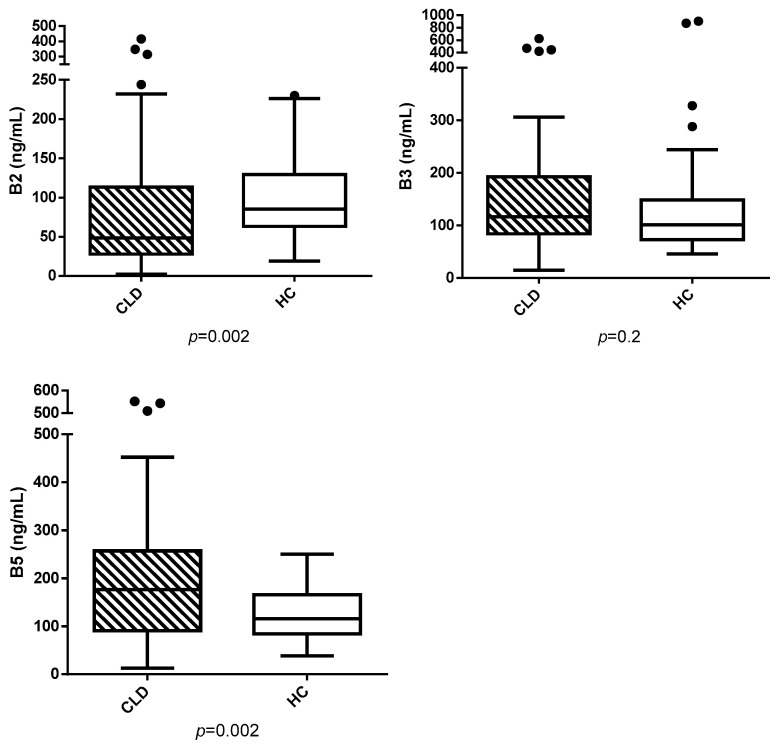
Serum vitamins B2, B3, and B5 (ng/mL) in dogs with chronic liver disease (CLD) and healthy controls (HC). Outliers are shown as black dots.

Vitamin B2 was significantly lower in CLD dogs compared to HC dogs (median 48.4 ng/mL (2.2–416) vs. 85.5 ng/mL (18.9–230), *p* = 0.002). Conversely, B3 levels did not differ significantly between the two groups (median 116.5 ng/mL (15–624) vs. 101 ng/mL (46–906), *p* = 0.25). However, B5 was significantly higher in CLD dogs (median 176.5 ng/mL (13–552) vs. 116.1 ng/mL (38.6–310), *p* = 0.002). Results are reported in Figure 1. In CLD dogs, serum liver enzymes did not significantly correlate with B2, B3, and B5 serum levels, with all *p* > 0.05.

Among dogs with CLD, differences were investigated between patients with and without biliary involvement (BTD versus non-BTD), with no significant differences observed in serum concentrations of vitamins B2 (*p* = 0.57), B3 (*p* = 0.37), and B5 (*p* = 0.39) between the two groups. According to the chronic intestinal involvement, no significant difference in serum vitamin B2 (*p* = 0.7), B3 (*p* = 0.12), and B5 (*p* = 0.25) concentrations was observed between dogs with and without chronic ultrasonographic intestinal alterations.

## 4. Discussion

This is the first study aimed to evaluate vitamin B2, B3, and B5 in dogs with CLD. Riboflavin (B2) was significantly reduced in CLD dogs when compared to healthy controls. Riboflavin, in its cofactor forms flavin adenine dinucleotide (FAD) and flavin mononucleotide (FMN), has fundamental roles in energy metabolism, cellular antioxidant potential, and metabolic interactions with other micronutrients, including iron, vitamin B6, and folate [12]. The liver is the main storage organ for FAD and FMN, where they are bound to specific proteins critical for metabolic functions [13]. In humans, riboflavin deficiency impairs mitochondrial function and antioxidant defences, leading to increased oxidative stress and lipid accumulation in the liver. This can initiate or exacerbate liver diseases such as MASLD. Conversely, CLDs can disrupt riboflavin metabolism and reduce its absorption, leading to deficiency. This deficiency further impairs liver function, creating a vicious cycle. Thus, riboflavin deficiency not only contributes to the development of CLDs but is also a result of the impaired liver function associated with these conditions. Addressing riboflavin deficiency may, therefore, be a crucial aspect of preventing and managing chronic liver diseases [14]. In rat models, riboflavin deficiency combined with a high-fat diet synergistically exacerbates hepatic lipid accumulation both in vivo and in vitro, potentially through activation of the peroxisome proliferator-activated receptor gamma (PPARγ) pathway, implicating riboflavin as a contributing factor in the pathogenesis of MASLD [15]. Evidence from multiple preclinical studies further supports the hepatoprotective role of riboflavin. Pretreatment with riboflavin has been shown to attenuate liver injury by reducing serum alanine aminotransferase (ALT) and aspartate aminotransferase (AST) levels, suppressing neutrophil infiltration, and decreasing oxidative stress markers—effects mechanistically mediated by modulation of nitric oxide production and reactive oxygen species (ROS) [16]. In carbon tetrachloride-induced liver fibrosis, riboflavin supplementation significantly reduced collagen deposition, alleviated hepatic fibrosis, and restored mitochondrial function [17]. Similarly, in murine models of alcohol-induced liver injury, riboflavin administration led to reductions in hepatic transaminases, lipid accumulation, and pro-inflammatory markers, while also modulating gut microbiota composition, suggesting involvement of the gut–liver axis [18]. Moreover, restoration of riboflavin status in mice ameliorated hepatic oxidative stress and intestinal inflammation, and normalized gut microbial dysbiosis [19]. In dogs, riboflavin was found to be increased during chronic kidney disease [20]; however, no studies are currently available regarding its potential alteration during canine gastrointestinal diseases or CLDs.

Niacin (B3) is a water-soluble vitamin essential for energy metabolism, as it serves as a precursor for the coenzymes Nicotinamide Adenine Dinucleotide (NAD^+^) and Nicotinamide Adenine Dinucleotide Phosphate (NADP^+^). These coenzymes are involved in numerous enzymatic reactions, including those in glycolysis, the citric acid cycle, and oxidative phosphorylation. Additionally, NADP^+^ plays a role in anabolic processes such as fatty acid and cholesterol synthesis, as well as in maintaining cellular antioxidant functions [21].

The liver is central to niacin metabolism, converting dietary tryptophan into niacin via the kynurenine pathway [22]. In cases of liver disease, this conversion process may be impaired. However, the body has compensatory mechanisms to maintain niacin levels. Extrahepatic tissues, including the kidneys and immune cells, can contribute to NAD^+^ biosynthesis, potentially offsetting hepatic deficiencies [23,24]. Moreover, the body can obtain niacin directly from the diet, and adequate dietary intake may help preserve normal plasma levels even in the presence of liver dysfunction [25,26]. In human medicine, studies have shown that niacin supplementation can have beneficial effects on liver conditions such as MASLD [27,28]. Niacin has been observed to inhibit hepatic fat accumulation, reduce oxidative stress, and decrease inflammation by modulating pathways like diacylglycerol acyltransferase 2 activity and Nicotinamide Adenine Dinucleotide Phosphate—Reduced Form (NADPH) oxidase activity [27,28,29,30,31]. These effects suggest that niacin plays a protective role in liver health. In dogs, the effect of niacinamide on high-density lipoprotein (HDL) metabolism was investigated in obese and insulin-resistant dogs. The study demonstrated that nicotinic acid significantly accelerated the turnover of HDL cholesteryl esters, indicating enhanced reverse cholesterol transport. This effect was independent of changes in HDL cholesterol concentrations, suggesting a functional improvement in HDL dynamics [32]. These findings highlight the potential of nicotinic acid as a therapeutic agent to improve lipid metabolism and reduce cardiovascular risk in insulin-resistant states. While direct data on serum niacin concentrations in dogs with hepatic disorders are currently lacking, the mechanisms observed in human studies may offer insights. The body’s ability to maintain niacin levels through dietary intake and extrahepatic synthesis could explain the lack of significant differences observed in this study between healthy and CLD dogs. Further research is needed to explore these mechanisms in dogs and to determine the clinical relevance of niacin in veterinary hepatology.

Pantothenic acid (B5) is a water-soluble vitamin that serves as a precursor for the synthesis of coenzyme A (CoA), a critical coenzyme involved in numerous biochemical reactions. CoA plays a pivotal role in the metabolism of carbohydrates, lipids, and proteins, facilitating the synthesis and oxidation of fatty acids, the citric acid cycle, and the biosynthesis of cholesterol and acetylcholine. The liver, being a central metabolic organ, is heavily involved in these processes, and, thus, pantothenic acid is integral to hepatic function [33,34,35]. Studies evaluating serum pantothenic acid in dogs show that it can be quantitatively tracked, and dietary modifications reliably modulate levels. In deficiency models, serum B5 may drop during nutritional restriction and weight loss nutritional programs [36]. In disease contexts like chronic kidney disease, serum B5 remains relatively stable [20]. The B5 levels in CLD dogs have never been evaluated before. The observation of elevated vitamin B5 (pantothenic acid) levels in hepatopathic dogs compared to healthy controls may be attributed to several physiological and pathological mechanisms. In human medicine, studies on vitamin B5 levels in chronic liver disease are also limited. Some research suggests that liver dysfunction can lead to altered vitamin metabolism, but specific data on pantothenic acid are scarce. The lack of comprehensive studies makes it challenging to draw direct comparisons or establish definitive conclusions regarding its levels in hepatic conditions. One possibility is that liver dysfunction impairs the utilization and conversion of pantothenic acid into CoA, leading to its accumulation in the bloodstream. Hepatocellular damage might reduce the expression or activity of enzymes required for CoA synthesis, resulting in decreased intracellular CoA levels and compensatory increases in circulating pantothenic acid [37]. Another consideration is the alteration of hepatic transport mechanisms. Liver disease can disrupt the normal uptake and storage of vitamins, potentially causing an overflow of pantothenic acid into the circulation [38]. Additionally, cholestasis, a common feature in certain hepatic conditions, might impair the excretion of water-soluble vitamins, contributing to elevated plasma levels [8,37].

The lack of correlation between serum levels of vitamins B2, B3, and B5 and hepatic enzyme activities may indicate that the regulation of these vitamins’ metabolic pathways occurs independently of the degree of hepatocellular damage reflected by circulating enzymatic markers. Indeed, liver enzymes are primarily indicators of cell injury or leakage, rather than direct measures of functional capacity or metabolic integrity. It should also be emphasized that in the context of chronic liver disease, serum enzyme activities are not always proportional to the severity, stage, or histological extent of the underlying hepatic pathology. Enzyme levels may fluctuate due to episodic cellular injury, individual variability, or compensatory mechanisms, thus limiting their utility as reliable proxies of overall disease burden [39,40]. Consequently, the absence of an association with vitamin status might reflect both the multifactorial regulation of B-vitamin metabolism—potentially involving extrahepatic tissues—and the inherent limitations of enzymatic markers in accurately capturing the complexity of chronic hepatopathies.

In this study, patients presenting with unequivocal signs of hepatic insufficiency, such as hyperammonemia or elevated preprandial bile acids, were excluded. While this approach prevented the evaluation of vitamin levels in dogs with overt liver failure, normal ammonia and bile acid concentrations cannot exclude the presence of certain grades of liver dysfunction. Consequently, we were unable to investigate, nor can we exclude, the potential role of hepatic function in regulating water-soluble vitamin metabolism within the included population. Future studies should, therefore, examine whether variations in vitamin status occur across different categories of hepatic disease, including conditions such as portosystemic shunts or portal vein hypoplasia, to better understand the relationship between hepatic function and vitamin homeostasis.

The presence of biliary involvement does not appear to be associated with variations in serum concentrations of these water-soluble vitamins. This observation may be explained by the differing physiological requirements for bile in the metabolism of water-soluble versus fat-soluble vitamins [41]. While the absorption and enterohepatic circulation of fat-soluble vitamins are heavily dependent on bile secretion and flow, water-soluble vitamins—such as B2, B3, and B5—are less reliant on biliary processes. Consequently, even in the presence of biliary disease, the intestinal uptake and systemic availability of water-soluble vitamins may remain largely unaffected. Therefore, the lack of association between biliary involvement and serum concentrations of these vitamins likely reflects both their relative independence from biliary function and the existence of multiple redundant mechanisms maintaining their systemic levels. Although serum deficiencies of vitamins B2, B3, and B5 have not yet been clearly documented in dogs with chronic enteropathy, as has been reported in humans [42], it cannot be excluded that, among dogs with CLD, the subset of animals presenting with ultrasonographic intestinal alterations may have introduced a potential bias. This raises the possibility that the observed results could be influenced not only by the presence of liver disease but also by concurrent intestinal pathology, thereby complicating the attribution of changes in vitamin status solely to hepatopathy.

This study should be interpreted in light of its limitations. Not all dogs with CLD underwent hepatic histopathological evaluation, and, therefore, a comprehensive characterization of liver disease was not always possible. Consequently, the CLD group may encompass considerable internal variability depending on the type and severity of the underlying condition. In addition, not all healthy dogs received an abdominal ultrasonographic examination; hence, it cannot be excluded that some control animals may have harbored subclinical hepatointestinal alterations, despite unremarkable hematobiochemical findings and absence of clinical signs.

## 5. Conclusions

This preliminary study is the first to evaluate serum B2, B3, and B5 in dogs with CLD. The findings suggest that riboflavin deficiency in CLD dogs warrants consideration due to its role in metabolic pathways and potential hepatoprotective effects, particularly concerning lipid dysregulation and oxidative stress. This study found no significant difference in vitamin B3 levels and an unexpected increase in vitamin B5 levels in hepatopathic dogs compared to healthy controls. These findings highlight possible alterations in vitamin metabolism due to liver dysfunction and underscore the need for further research to elucidate underlying mechanisms and clinical relevance. Neither biliary involvement nor variations in hepatic enzyme activities were associated with serum concentrations of vitamins B2, B3, and B5, suggesting that their status may be partially independent of hepatocellular injury or biliary physiology.

## Figures and Tables

**Table 1:** MS main parameters.

	Q1 → Q3	DP	EP	CE	CXP
Vitamin B2	377.2 → 172.2	130	9	48	3.7
	377.2 → 196.1	130	9	52	4.6
	377.2 → 243.2	130	9	31	6
Vitamin B2-^13^C_4_,^15^N_2_	383.1 → 175.1	130	9	48	3.7
	383.1 → 200.0	130	9	52	4.6
	383.1 → 249.0	130	9	31	6
Vitamin B3	123.2 → 52.9	90	9	42	3.9
	123.2 → 80.0	90	9	27	6.3
	123.2 → 96.2	90	9	25	8
Vitamin B3-^13^C_6_	129.2 → 57.0	90	9	42	3.9
	129.2 → 85.0	90	9	27	6.3
	129.2 → 101.0	90	9	25	8
Vitamin B5	220.3 → 71.9	75	9.3	33	5.4
	220.3 → 90.0	75	9.3	20	8.1
	220.3 → 96.0	75	9.3	27	7.2
Vitamin B5 ^13^C_3_,^15^N	224.0 → 75.8	75	9.3	33	5.4
	224.0 → 94.1	75	9.3	20	8.1
	224.0 → 126.3	75	9.3	30	7.2

**Table 2:** Descriptive statistics of serum hepatic enzymes, total bilirubin (TotBil), cholesterol (Chol), triglycerides (Trig), total protein (TP), and albumin (Alb) in CLD (chronic liver disease) dogs (median and range).

Parameter	CLD Dogs (n = 66)	Reference Range
ALP (U/L)	782 (31–2100)	45–250
GGT (U/L)	14.5 (0.1–184)	2–11
AST (U/L)	44.5 (18–909)	15–40
ALT (U/L)	139 (5–1170)	20–70
Tot Bil (mg/dL)	0.2 (0.1–27.76)	0.07–0.3
TP (g/dL)	6.5 (4.3–8.8)	5.8–7.8
Alb (g/dL)	3.4 (2–4.6)	2.6–4.1
Chol (mg/dL)	285 (115–541.6)	120–280
Trig (mg/dL)	100 (49–213)	25–90

## Data Availability

The data presented in this study are available on request from the corresponding author due to ongoing research.

## Abbreviations

The following abbreviations are used in this manuscript:

MASLD	Metabolic Dysfunction-Associated Steatotic Liver Disease
CLD	Chronic liver disease
ALP	Alkaline phosphatase
GGT	Gamma-glutamyl transferase
ALT	Alanine aminotransferase
AST	Aspartate aminotransferase
HC	Healthy controls
BTD	Biliary tract disease
FAD	Flavin adenine dinucleotide
FMN	Flavin mononucleotide
PPARγ	Peroxisome proliferator-activated receptor gamma
ROS	Reactive oxygen species
NAD^+^	Nicotinamide Adenine Dinucleotide
NADP^+^	Nicotinamide Adenine Dinucleotide Phosphate
NADPH	Nicotinamide Adenine Dinucleotide Phosphate—Reduced Form
HDL	High-density lipoprotein
CoA	Coenzyme A
